# Individual Placement and Support in der psychiatrischen Versorgung: Evaluation klinischer Routinedaten mittels eines retrospektiven Chart-Reviews

**DOI:** 10.1007/s00115-024-01706-5

**Published:** 2024-07-18

**Authors:** Dorothea Jäckel, Karolina Leopold, Andreas Bechdolf

**Affiliations:** 1 Kliniken für Psychiatrie, Psychotherapie und Psychosomatik mit FRITZ am Urban und soulspace, Vivantes Klinikum Am Urban und Vivantes Klinikum im Friedrichshain, Dieffenbachstr. 1, 10967 Berlin, Deutschland; 2 Klinik für Psychiatrie und Psychotherapie, CCM, Charité – Universitätsmedizin Berlin, corporate member of Freie Universität Berlin und Humboldt-Universität zu Berlin, Berlin, Deutschland; 3 Klinik für Psychiatrie und Psychotherapie, Universität Carl Gustav Carus, Dresden, Deutschland; 4 Deutsches Zentrum für psychische Gesundheit (DZPG) Standort Berlin-Potsdam, Berlin, Deutschland

**Keywords:** Klinische Versorgung, Exklusion, Return to work, Allgemeiner Arbeitsmarkt, Teilhabe, Clinical healthcare, Exclusion, Return to work, Open labor market, Participation

## Abstract

**Hintergrund:**

Menschen mit schweren psychischen Erkrankungen sind häufig aus dem Arbeitsleben exkludiert und wünschen im Rahmen der psychiatrischen Behandlung Unterstützung bei der (Wieder)aufnahme einer Arbeit auf dem allgemeinen Arbeitsmarkt. Individual Placement and Support (IPS) ist ein wirksames Verfahren, Arbeit auf dem allgemeinen Arbeitsmarkt zu finden und zu halten. Ziel der Arbeit war die Ermittlung der Eingliederungsraten von IPS auf den allgemeinen Arbeitsmarkt im akuten und postakuten psychiatrischen Setting sowie die Identifikation von Patienten‑, Setting- und Programmmerkmalen, die mit der (Wieder)aufnahme von Arbeit assoziiert waren.

**Methodik:**

Retrospektiver Chart-Review (RCR) klinischer Routinedaten zwischen 2016 und 2021. Rating der IPS-Programmtreue mithilfe der IPS-Fidelity-Scale.

**Ergebnisse:**

Insgesamt 375 Patienten nahmen mit mindestens vier Terminen am IPS teil. Die (Wieder)eingliederungsrate auf den allgemeinen Arbeitsmarkt betrug 51,7 %. Eine kürzere Zeitspanne zum letzten Arbeitstag, die Diagnosen F1, F2, F3 (vs. F4), ein Wechsel des Behandlungssettings und IPS in der psychiatrischen Institutsambulanz (PIA), IPS-Fidelity sowie die Anzahl der IPS-Coachings waren positiv mit der (Wieder)aufnahme von Arbeit korreliert.

**Schlussfolgerungen:**

Die Umsetzung von IPS in der klinischen Versorgung ist möglich und führt zu hohen Eingliederungsraten auf dem allgemeinen Arbeitsmarkt. Ein früher Beginn von IPS während der klinischen Behandlung kann die soziale Inklusion fördern.

## Abstract (en)

**Background:**

People with severe mental illnesses are often excluded from working life and would like support to (re)enter the general labor market as part of the psychiatric treatment. Individual placement and support (IPS) is an effective method of finding and retaining work in the general labor market. The aim of the study was to determine the integration rates of IPS into the general labor market in acute and postacute psychiatric settings, identifying patient, setting and program characteristics associated with (re)entering work.

**Method:**

A retrospective chart review (RCR) of routine clinical data between 2016 and 2021 was carried out. The IPS program adherence was rated using the IPS fidelity scale.

**Results:**

A total of 375 patients participated in the IPS with at least 4 appointments. The (re)integration rate into the general labor market was 51.7%. A shorter time period to the last working day, diagnosis of F1, F2, F3 (vs. F4), change of treatment setting and IPS in the psychiatric institute outpatient department (PIA), IPS fidelity and the number of IPS coaching sessions were positively correlated with (re)integration into work.

**Conclusion:**

The implementation of IPS in clinical mental healthcare is possible and leads to high integration rates in the open labor market. An early start of IPS during the clinical treatment can promote social inclusion.

## Hintergrund und Fragestellung

Menschen mit schweren psychischen Erkrankungen sind häufig aus dem Arbeitsleben ausgeschlossen [1, 2] und viele von ihnen möchten (wieder) auf dem allgemeinen Arbeitsmarkt arbeiten [3]. Das Risiko für ein erschwertes „return to work“ steigt bereits nach 6 Monaten ohne Arbeit sprunghaft an und eine Rückkehr gelingt häufig nicht mehr, aufgrund von Selektionsprozessen am Arbeitsmarkt sowie personenseitigen Faktoren [4, 5]. Eine moderne Psychiatrieversorgung strebt an, derartigen Chronifizierungsprozessen entgegenzuwirken und Teilhabe im Sinne der UN-Behindertenrechtskonvention schon während der akuten und postakuten psychiatrischen Behandlung zu fördern [6].

Der derzeit wirksamste Ansatz für Menschen mit schweren psychischen Erkrankungen, wieder in Arbeit zu kommen, stellt Individual Placement and Support (IPS) dar. IPS konnte weltweit in mittlerweile 38 randomisiert kontrollierten Studien eine etwa doppelte Wirksamkeit im Vergleich zu üblichen Eingliederungsmaßnahmen zeigen. Die Eingliederungsraten in den ersten Arbeitsmarkt betragen durchschnittlich 60 % (außerhalb Europas) und 44 % (Europa); [7, 8]. In der Routineversorgung erreicht IPS im Mittel Eingliederungsraten von 42 % [9]. Internationale Studien zeigen, dass Alter, Geschlecht [10], frühere Arbeitserfahrungen [11], psychiatrische Diagnosen [10], Dauer des IPS-Coachings [11] sowie die IPS-Fidelity [12] mit den Eingliederungsraten korrelieren. In den aktuellen S3-Leitlinien wird IPS mit dem höchsten Empfehlungsgrad empfohlen [13] und die Deutsche Gesellschaft für Psychiatrie, Psychotherapie und Neurologie (DGPPN) befürwortet in ihrem Positionspapier eine Verankerung von IPS in der psychiatrischen Versorgung in Deutschland [14]. Erste Studien zeigen, dass IPS auch im deutschen Versorgungssystem anwendbar [15], wirksam und kosteneffektiv ist [16].

Im Vivantes Klinikum Am Urban, Berlin wurde IPS im April 2016 eingeführt und steht allen Patienten in (teil)stationärer psychiatrischer Behandlung und der psychiatrischen Institutsambulanz (PIA) zur Verfügung, die Unterstützung bei der (Wieder)aufnahme einer Arbeit wünschen. Die Begleitung durch die IPS-Coaches erfolgt settingübergreifend [17]. IPS basiert auf acht pragmatischen Prinzipien:

1. zügige Jobsuche,
2. nach Arbeitsstellen auf dem allgemeinen Arbeitsmarkt,
3. Vernetzung mit dem regionalen Arbeitsmarkt,
4. Ausrichtung der Jobsuche an den Präferenzen der Teilnehmenden,
5. keine Person wird ausgeschlossen, die an der Aufnahme einer Arbeit interessiert ist („zero exclusion“),
6. zeitlich unbefristete Begleitung nach Arbeitaufnahme,
7. finanzielle Beratung und
8. Integration in die psychiatrische Behandlung [18].

Die Klinik für Psychiatrie, Psychotherapie und Psychosomatik des Vivantes Klinikum Am Urban in Berlin hat einen Versorgungsauftrag für den Bezirk Friedrichshain-Kreuzberg, verfügt über 170 stationäre und 21 stationsäquivalente, 4 jugendpsychiatrische, 54 tagesklinische Behandlungsplätze und versorgt ca. 2400 Patienten in der psychiatrischen Institutsambulanz (PIA).

Nach Kenntnis der Autorinnen liegen in Deutschland keine Studien zur Praktikabilität, Umsetzung und Wirksamkeit von IPS in der klinischen Routineversorgung bei einem größeren Patientenkollektiv vor. Ziele der vorliegenden Studie war es, (Wieder)eingliederungsraten von Patienten zu ermitteln, die im akuten und postakuten psychiatrischen Setting am IPS teilnahmen, und Erkenntnisse über Patientenmerkmale, Inanspruchnahme von IPS sowie die IPS-Programmtreue zu gewinnen.

## Studiendesign und Untersuchungsmethoden

### Datengrundlage

In einem retrospektiven Chart-Review (RCR) wurden Patientenfälle untersucht, die am IPS im Rahmen ihrer (teil)stationären und/oder ambulanten psychiatrischen Behandlung im Klinikum Am Urban in Berlin teilnahmen. Aus dem Klinikinformationssystem (KIS) wurden die Patientenfälle extrahiert, die im Zeitraum vom 01.04.2016 bis zum 31.03.2021 im IPS vorstellig wurden und mindestens 4 Termine während 6 Monaten wahrgenommen haben. Dieser Cut-off wurde gewählt, um Fälle auszuschließen, die lediglich Informationen zum IPS oder eine Kurzberatung wünschten. Neben den Patientenstammdaten (u. a. Alter, Geschlecht, Diagnose, Behandlungssetting) liegt im KIS die fortlaufende Dokumentation des arbeitsbezogenen Outcomes durch die IPS-Coaches vor. Der Datenexport erfolgte im März 2024. Die Berichterstattung folgt den Kriterien des RECORD-Statements [19].

#### Programm-Fidelity

Die Programm-Fidelity von IPS wurde mit der 25 Items umfassenden IPS-Fidelity-Scale im Selbstassessment erhoben, die den Umsetzungsgrad des IPS-Programms auf drei Dimensionen abbildet: 1. Personal, 2. Organisation und 3. spezifische Dienstleistungen im IPS. Jedes Item wird auf einer 5‑stufigen Likert-Skala eingeschätzt, die von 1 (keine Implementierung) zu 5 (vollständige Implementierung) reicht. Der Summenscore differenziert vier Kategorien: 115 bis 125 Punkte zeigen eine „modellgetreue“, 100 bis 114 Punkte eine „gute“, 74 bis 99 Punkte eine „moderate“ Fidelity an. Scores ≤73 Punkten entsprechen nicht dem IPS-Modell [12]. Die Fidelity wird jährlich zu Beginn des 2. Quartals erhoben.

Primäre Zielgröße der Studie war die (Wieder)aufnahme einer kompetitiven Arbeit operationalisiert über das Kriterium, im Beobachtungszeitraum von 66 Monaten mindestens einen Tag auf dem allgemeinen Arbeitsmarkt zu arbeiten. Dabei handelt es sich um das international anerkannte primäre Outcomekriterium für Studien und Metaanalysen zum IPS [8].

#### Variablendefinitionen

Die Prädiktoren der (Wieder)aufnahme einer kompetitiven Arbeit auf dem allgemeinen Arbeitsmarkt zur Baseline waren:

- Alter,
- Gender (männlich, weiblich; [10]),
- Zeitabstand zum letztem Arbeitstag (0–6 Monate, 6–36 Monate, länger als 36 Monate; [11]) ermittelt durch Differenz des Vorstellungsdatums im IPS (mm/yyyy) und dem letzten Arbeitstag (mm/yyyy),
- psychiatrische Diagnose gemäß International Statistical Classification of Diseases and Related Health Problems 10 (ICD-10; [10]),
- IPS-Fidelity [12].
- Im Beobachtungszeitraum:
  - Wechsel des Behandlungssettings in die PIA (ja/nein),
  - Anzahl der IPS-Coachings [11].

Als abhängige Variable wurde die (Wieder)aufnahme einer kompetitiven Arbeit auf dem allgemeinen Arbeitsmarkt für mindestens einen Tag (ja/nein) festgelegt.

Die soziodemografischen Angaben (Alter, Gender), die Haupt- und Nebendiagnosen, das Vorstellungsdatum im IPS sowie der Wechsel des Behandlungssettings/PIA wurden der KIS-Basisdokumentation entnommen und in die Datenbank aufgenommen. Aus der Verlaufsdokumentation der IPS-Coaches im Klinikinformationssystem stammten die Angaben zur Inanspruchnahme von IPS operationalisiert über die Anzahl der IPS-Coachings sowie Angaben zur Arbeitssituation der Patienten. Mit dem Export aus dem System wurden sämtliche personenbezogenen Angaben gelöscht, die einzelnen Datensätze fortlaufend nummeriert, sodass sie in anonymisierter und unrekonstruierbarer Form vorlagen. Kein Merkmal weist eine Häufigkeit ≤5 auf. Abschließend wurde jeder Datensatz mit dem für das jeweilige Jahr erhobene IPS-Fidelity-Score (gut vs. moderat) als zusätzliche Prädiktorvariable ergänzt.

#### Statistische Analysen

In der deskriptiven Statistik wurden absolute (*n*) und prozentuale Angaben (%), das arithmetische Mittel (M), die Standardabweichung (SD), der Median (Md) und der Range berechnet. Um den Einfluss ausgewählter Variablen auf das Merkmal „(Wieder)aufname einer Arbeit“ zu ermitteln, wurde eine logistische Regression gerechnet. Die Beurteilung der Modellgüte basiert auf dem Bestimmtheitsmaß Nagelkeres R^2^. Es wurde eine Complete-cases-Analyse durchgeführt. Fehlende Werte im Prädiktor „Zeitabstand zum letztem Arbeitstag“ betrugen 13,6 %. In der Regression wurden die mittels Benjamini-Hochberg False Discovery Rate (FDR) korrigierten kritischen *p*-Werte verwendet. Als statistisch signifikant wurde ein *p*-Wert von <0,05 festgelegt. Die statistischen Analysen wurden mit JMP® 17.2 (SAS Institute, Cary, NC, USA) durchgeführt.

## Ergebnisse

### Stichprobe

Im Beobachtungszeitraum zwischen 04/2016 und 09/2021 nahmen 375 Patienten mit mindestens 4 Terminen am IPS teil. Die Beobachtungsdauer betrug zwischen 6 und 66 Monaten. Die Merkmale des Patientenkollektivs sind in Tab. 1 aufgeführt.

**Tab. 1:** Beschreibung des Patientenkollektivs (*N* = 375)

*Alter*, MW (SD) Md (Range)	37,7 (10,6) 37 (19–63)
*Gender*: weiblich, *n* (%)	225 (60)
*Diagnosegruppe (Hauptdiagnose), n* (%)	
F1.x	9 (2,5)
F2.x	89 (23,7)
F3.x	197 (52,5)
F4.x	42 (11,2)
F6.x	38 (10,1)
*Komorbidität* ^a^, *n* (%)	173 (46,1)
*Substanzstörung* ^b^, *n* (%)	64 (17,1)
*Zeitabstand zum letzten Arbeitstag*^c^, *n* (%)	
0–6 Monate	197 (60,8)
6–36 Monate	92 (28,4)
>36 Monate	35 (10,8)
*Wechsel des Behandlungssettings/PIA, n* (%)	313 (83,5)
*IPS-Programm Fidelity, n* (%)	
„gut“	240 (64)
„moderat“	135 (36)
*IPS-Coaching Kontakte*, MW (SD) Md (Range)	13,6 (14) 8 (4–111)
*(Wieder)aufnahme einer Arbeit, n* (%)	194 (51,7)

*Md *Median, *MW* Mittelwert, *IPS* Individual Placement and Support, *PIA *psychiatrische Institutsambulanz, *SD* Standardabweichung

^a^Vorliegen mind. einer psychiatrischen Nebendiagnose

^b^F1x.2 oder F1x.2, ohne F17 (Tabak)

^c^Missings *n* = 51

Insgesamt 84 % der Patienten nahmen mit einem Wechsel vom (teil)stationären Setting in die PIA oder durchgängig im Rahmen ihrer PIA-Behandlung am IPS teil. 16 % nahmen IPS nur im Rahmen der (teil)stationären Behandlung in Anspruch. Die Programm- bzw. Umsetzungstreue erhoben mit der IPS-Fidelity-Scale konnte innerhalb des ersten Jahres (2016) von 89 (moderat) auf 104 Punkte (von 125 möglichen Punkten) gesteigert und bis Anfang 2020 gehalten werden. Danach sank die Fidelity auf ein moderates Niveau mit 88 Punkten ab und erreicht 2021 erneut ein gutes Niveau mit 103 Punkten. 64 % der Patienten haben unter der Bedingung einer guten Programmtreue mit dem IPS begonnen.

### Prädiktoren für die (Wieder)aufnahme einer Arbeit

Als potenzielle prädiktive Faktoren für die (Wieder)aufnahme einer Arbeit stellten sich bei der Regressionsanalyse 4 der 6 Variablen als signifikant heraus:

- eine kürzere Zeitspanne (in Monaten) zum letzten Arbeitstag,
- eine ICD-10-Diagnose mit F1, F2 oder F3 (vs. F4),
- Wechsel des Behandlungssettings/PIA (vs. nur [teil]stationäre Behandlung),
- eine „gute“ IPS-Fidelity (vs. eine „moderate“).

Die Ergebnisse der logistischen Regression sind in Tab. 2 abgebildet.

**Tab. 2:** Prädiktorvariablen für die (Wieder)aufnahme einer Arbeit (*n* = 324)

	Odds Ratio	*p*	95 %-Konfidenzintervall
*Alter*	0,98	0,102	0,96–1,00
*Gender* (w)^a^	0,39	0,354	0,14–1,15
*Diagnosegruppe*^b^			
F1	9,41	0,019	1,44–61,26
F2	3,98	0,003	1,59–9,96
F3	3,44	0,003	1,51–7,87
F6	1,52	0,459	0,50–4,60
*Zeitabstand zum letzten Arbeitstag*^c^			
0–6 Monate	8,20	<0,0001	3,06–21,97
6–36 Monate	3,44	0,018	1,24–9,55
*Wechsel des Behandlungssettings/PIA*^d^	5,14	<0,0001	2,43–10,85
*IPS Fidelity „gut“*^e^	1,90	0,016	1,13–3,21

*IPS* Individual Placement and Support, *PIA *psychiatrische Institutsambulanz

^a^Referenz: Gender männlich

^b^Referenz: Diagnosegruppe F4

^c^Referenz: > 36 Monate

^d^Referenz: nur (teil)stationäre Behandlung

^e^Referenz: IPS-Fidelity „moderat“

Das Regressionsmodell war statistisch signifikant χ^2^ (10) = 75,52, *p* < 0,001, mit einer akzeptablen Varianzaufklärung von Nagelkerkes R^2^ 0,28 und klassifizierte 70 % der (Wieder)aufnahmen einer Arbeit korrekt.

## Diskussion

Ziel der Studie war es, anhand eines RCR die Eingliederungsraten von IPS auf den ersten Arbeitsmarkt in der psychiatrischen Routineversorgung zu untersuchen. In der bisher deutschlandweit größten untersuchten Population zeigen die Ergebnisse, dass IPS in der klinischen Versorgung an einem breiten Diagnosespektrum umgesetzt werden kann und bei 51,7 % der 375 Patienten zu der angestrebten (Wieder)eingliederung auf dem allgemeinen Arbeitsmarkt führt. Die Resultate sind vergleichbar mit denen in einer Metaanalyse berichteten durchschnittlichen Eingliederungsraten in der Routineversorgung von durchschnittlich 43 % (Range 18 %–82 %) im IPS vs. 18 % bei konventionellen Eingliederungsmaßnahmen [9]. Weder Alter noch Gender erwiesen sich in unserer Studie als prädiktiv für das Outcome; sowohl jüngere wie ältere Patienten, Frauen wie Männer profitierten vom IPS. Andererseits finden Studien zum IPS mit jungen Erwachsenen deutlich höhere Eingliederungsraten bis zu 90 % [16, 20], was Anlass zu vorsichtigem Optimismus gibt, insbesondere bei dieser Personengruppe durch frühzeitige IPS-Angebote der drohenden sozialen Exklusion wirksam begegnen zu können.

Der Zeitabstand zum letzten Arbeitstag war mit der späteren Arbeitsaufnahme assoziiert: Je kürzer der letzte Arbeitstag zurücklag, desto höher war die Wahrscheinlichkeit (wieder) in Arbeit zu kommen. Diese Resultate zeigen die hohe Zweckdienlichkeit, arbeitsbezogene Teilhabe bereits in der Klinik anzugehen, um den Unterstützungsbedarf der Patienten frühzeitig in der akuten oder postakuten Behandlungsphase, leicht zugänglich und entlang der individuellen Motivation, (wieder) zu arbeiten, aufzugreifen [3, 21, 22]. Das bedeutet auch, dass das im (teil)stationären Setting begonnene IPS nicht mit der Entlassung endet, sondern im Rahmen der PIA-Behandlung fortgesetzt wird. Dieser Settingwechsel wirkt sich günstig auf die (Wieder)eingliederung aus, wie das Regressionsmodell zeigte. Die IPS-Fidelity und die Anzahl der IPS-Coachings korrelierten ebenfalls positiv mit der (Wieder)aufnahme einer Arbeit, was darauf hindeutet, dass die Prinzipien von IPS generell und die auf Langfristigkeit angelegte Unterstützung im IPS im Besonderen umgesetzt wurden. Diese Zusammenhänge entsprechen den Befunden in den Referenzstudien, die bessere Eingliederungsraten in Verbindung mit einer hohen Programmtreue gefunden haben [23, 24]. Hinsichtlich der Diagnosen profitierte die Patientengruppen mit F1-, F2- und F3-Diagnosen mehr vom IPS als denjenigen mit F4, was sich mit den Ergebnissen aus der Schweiz von Richter et al. [9] deckt, die hingegen bei der Patientengruppe mit Erkrankungen aus dem schizophrenen Formenkreis einen massiven Anmeldungsrückgang verzeichnen. Es sind jedoch insbesondere Menschen mit schweren psychischen Erkrankungen, die vom IPS profitieren und weniger diejenigen mit „common mental disorder“ (CMD; F4-Diagnosen; [10]). Ein Grund könnte darin liegen, dass motivationale Konflikte hinsichtlich des beruflichen Wiedereinstiegs, wie sie bei Patienten mit CMD in relevantem Umfang vorzuliegen scheinen, durch IPS nicht ausreichend adressiert werden [25]. Hier besteht Forschungsbedarf nach Interventionen, die dabei helfen, dass das anfängliche Interesse („zero exclusion“), wieder einer Arbeit auf dem allgemeinen Arbeitsmarkt nachzugehen, tatsächlich in einer Arbeitsaufnahme mündet. Daran schließt sich die praktisch relevante Frage an, wie lange IPS-Coaches ihr an den IPS-Prinzipien orientiertes Engagement optimalerweise anbieten sollten, wenn passende Stellenangebote von den Patienten nicht genutzt, Krankschreibungen nicht enden und sich der (Wieder)einstieg in Arbeit immer wieder hinauszögert.

Seit rund 10 Jahren wird IPS an einzelnen psychiatrischen Kliniken in Deutschland genutzt [15] und das Thema der arbeitsbezogenen Teilhabe erhält zunehmend Aufmerksamkeit im klinischen Versorgungsalltag [14, 26]. Aktuelle Forschungsprojekte haben das Thema Arbeit bereits in ihren Fokus genommen wie z. B. SEEearly [27] LIPSY [28] RTW PIA [29] und RETURN [30]. Zukünftigen Studien ist vorbehalten, IPS-Programme mit konkreten Kosten zu hinterlegen, diese mit den zeitlich befristeten und teuren Rehabilitationsmaßnahmen zu vergleichen, um zu dezidierten Aussagen hinsichtlich des Kosten-Nutzen-Verhältnisses von IPS zu gelangen. Erste Befunde aus dem deutschsprachigen Raum liegen hierzu bereits vor [16, 31] und geben Hinweise darauf, dass IPS sowohl wirksamer als auch kosteneffizienter ist.

### Stärken der Studie

- Die Studie wurde über einen langen Beobachtungszeitraum von 66 Monaten durchgeführt und schloss ein großes Kollektiv von Patienten (*N* = 375), die am IPS teilgenommen haben, ein.
- Sowohl personen-, setting- und programmbezogene Faktoren konnten in die Analyse einbezogen werden, was zu einer akzeptablen Varianzaufklärung führte.
- Es wurden Routinedaten eines Krankenhauses mit Versorgungsauftrag genutzt, die die (Wieder)aufnahme einer Arbeit prädizieren und weitgehend in Übereinstimmung mit der aktuellen Studienlage stehen.

### Limitationen der Studie

- Einschränkungen ergeben sich aus dem Studiendesign einer retrospektiven Beobachtungsstudie, der Verwendung von Routinedaten, die nicht zu Forschungszwecken erhoben wurden und der mangelnden Kontrollgruppe von Patienten, die kein IPS in Anspruch nahmen. Offen bleibt derzeit, inwieweit die Ergebnisse auf andere Regionen und Kliniken übertragbar sind.
- Einschränkungen ergeben sich weiterhin in Bezug auf das international etablierte primäre Wirksamkeitskriterium, die (Wieder)aufnahme einer Arbeit für mindestens einen Tag im Beobachtungszeitraum, das zwar die Voraussetzung einer (Wieder)eingliederung abbildet, für weiterreichende Analysen wie die der Nachhaltigkeit sozialer Teilhabe jedoch nicht suffizient ist.

## Fazit für die Praxis

- Die Implementierung von Individual Placement and Support (IPS) in die klinische Versorgung ist möglich und führt zu hohen Eingliederungsraten.
- Die Nutzung von IPS zeigt, dass der Bedarf von Patienten in der psychiatrischen Klinik nach einfacher Zugänglichkeit und langfristiger Unterstützung bei der (Wieder)aufnahme einer Arbeit auf dem allgemeinen Arbeitsmarkt besteht.
- IPS in der klinischen Versorgung sollte so konzipiert sein, dass es in der akuten oder postakuten psychiatrischen Behandlungsphase beginnt und im Anschluss nahtlos im Rahmen der PIA fortgesetzt wird.
- Ein frühzeitiger Beginn von IPS in oder nach der akutpsychiatrischen Krankheitsepisode kann helfen, einer Exklusion vom Arbeitsleben entgegenzuwirken.
